# Resting Heartbeat Complexity Predicts All‐Cause and Cardiorespiratory Mortality in Middle‐ to Older‐Aged Adults From the UK Biobank

**DOI:** 10.1161/JAHA.120.018483

**Published:** 2021-01-19

**Authors:** Lei Gao, Arlen Gaba, Longchang Cui, Hui‐Wen Yang, Richa Saxena, Frank A. J. L. Scheer, Oluwaseun Akeju, Martin K. Rutter, Men‐Tzung Lo, Kun Hu, Peng Li

**Affiliations:** ^1^ Department of Anesthesia Critical Care and Pain Medicine Massachusetts General Hospital Harvard Medical School Boston MA; ^2^ Medical Biodynamics Program Brigham and Women’s Hospital Boston MA; ^3^ Broad Institute of MIT and Harvard Cambridge MA; ^4^ Center for Genomic Medicine Massachusetts General Hospital Boston MA; ^5^ Division of Sleep Medicine Harvard Medical School Boston MA; ^6^ Division of Diabetes Endocrinology & Gastroenterology The University of Manchester Manchester UK; ^7^ Institute of Translational and Interdisciplinary Medicine and Department of Biomedical Sciences and Engineering National Central University Taoyuan Taiwan

**Keywords:** autonomic nervous system, complexity, distribution entropy, mortality, resting ECG, Biomarkers, Physiology

## Abstract

**Background:**

Spontaneous heart rate fluctuations contain rich information related to health and illness in terms of physiological complexity, an accepted indicator of plasticity and adaptability. However, it is challenging to make inferences on complexity from shorter, more practical epochs of data. Distribution entropy (DistEn) is a recently introduced complexity measure that is designed specifically for shorter duration heartbeat recordings. We hypothesized that reduced DistEn predicted increased mortality in a large population cohort.

**Method and Results:**

The prognostic value of DistEn was examined in 7631 middle‐older–aged UK Biobank participants who had 2‐minute resting ECGs conducted (mean age, 59.5 years; 60.4% women). During a median follow‐up period of 7.8 years, 451 (5.9%) participants died. In Cox proportional hazards models with adjustment for demographics, lifestyle factors, physical activity, cardiovascular risks, and comorbidities, for each 1‐SD decrease in DistEn, the risk increased by 36%, 56%, and 73% for all‐cause, cardiovascular, and respiratory disease–related mortality, respectively. These effect sizes were equivalent to the risk of death from being >5 years older, having been a former smoker, or having diabetes mellitus. Lower DistEn was most predictive of death in those <55 years with a prior myocardial infarction, representing an additional 56% risk for mortality compared with older participants without prior myocardial infarction. These observations remained after controlling for traditional mortality predictors, resting heart rate, and heart rate variability.

**Conclusions:**

Resting heartbeat complexity from short, resting ECGs was independently associated with mortality in middle‐ to older‐aged adults. These risks appear most pronounced in middle‐aged participants with prior MI, and may uniquely contribute to mortality risk screening.

**Nonstandard Abbreviations and Acronyms**
DistEn: distribution entropy
HRV: heart rate variability
NHS: National Health Service
RHR: resting heart rate
RMSSD: root mean square of successive differences
RRI: RR interval
TDI: Townsend deprivation index
UKB: UK Biobank

**Clinical Perspective**

**What Is New?**

- Distribution entropy—a measure of heartbeat complexity derived from a 2‐minute, resting ECG—predicts mortality independently of resting heart rate and heart rate variability in middle‐ to older‐aged adults.

**What Are the Clinical Implications?**

- These risks appear most pronounced in middle‐aged patients with prior myocardial infarction, and may uniquely contribute to routine mortality risk screening.
- Our findings have the potential to be scaled‐up to remote monitoring in wearable devices, and opens up a new avenue of research for resting heartbeat complexity as a vulnerability marker for stress reactivity.

With the exponential growth in noninvasive, passive monitoring of physiological function, there is burgeoning interest in extracting prognostic information from heart activity.^1^ Prior studies have relied on prolonged periods lasting hours^2^ to days,^1^ or depended on interventions such as exercise (heart rate recovery or exercise capacity) to derive prognostic information. However, exercise testing has not always been feasible because of contraindications in physical limitations or comorbidities. In fact, a meta‐analysis of large randomized controlled trials found questionable improvement in the prediction of health outcomes using exercise ECGs.^3^ Thus, convenient passive monitoring of resting heart activity that is not tightly coupled to a patient’s effort is becoming increasingly desirable.

In the past 2 decades, novel analytic tools derived from nonlinear dynamics have harnessed information imbedded in spontaneous physiological fluctuations; these are usually not obtainable through traditional, nonlinear signal processing approaches such as means/medians or frequency‐ and time‐domain analysis of heart rate variability (HRV). One of the predominant nonlinear methods is entropy, a measure of unpredictability or “complexity” of signals.^4^, ^5^, ^6^ Many have observed a loss/reduction of complexity in disease states,^6^, ^7^, ^8^, ^9^, ^10^ aging, frailty,^5^, ^6^, ^7^ and death.^11^, ^12^ Population screening tools that can guide clinical decisions demand convenience. Unfortunately, the study of complexity of physiological systems is data intensive. This is further exacerbated by the short time available in most standard clinical measurements of the heart, eg, during routine screening ECGs at rest.

A recently introduced distribution entropy (DistEn) measure considers a more integrated feature in the fluctuation patterns, thus allowing a reliable assessment of complexity from short recordings. It may fundamentally address the major limitation associated with short physiological recordings.^13^, ^14^ Recent studies have demonstrated its performance in quantifying the impact of diseases on physiological control.^13^, ^14^, ^15^, ^16^, ^17^ However, large‐scale longitudinal studies are required to determine the prognostic value of DistEn. We tested for the first time the association between DistEn derived from short, 2‐minute resting ECGs and mortality. We assessed whether this was independent of baseline comorbidities and traditional indicators of autonomic function, including RR interval (RRI), resting heart rate (RHR), conventional time‐domain HRV, and mean arterial pressure (MAP), in a large cohort from the UK Biobank (UKB).^18^, ^19^, ^20^

## Methods

### Study Participants

UKB is a large‐scale biomedical database and research resource containing genetic, lifestyle, and health information from half a million UK participants. Between late 2009 and 2010, 8039 participants from across the United Kingdom recruited to the UKB undertook a 2‐minute resting ECG.^21^ During this baseline assessment, participants also reported their demographics, lifestyle, medical conditions, and medications taken. Individuals were followed up until early 2018, the most up‐to‐date recording for mortality before data analysis on December 1, 2019. The UKB received National Research Ethics Approval and participants gave written informed consent. This study was conducted under the terms of UKB access for project 40556, and Partners HealthCare institutional review board approval. The data that support the findings of this study are available from the corresponding author on reasonable request.

### Preprocessing and Quality Control of Resting ECGs

**Figure 1:**
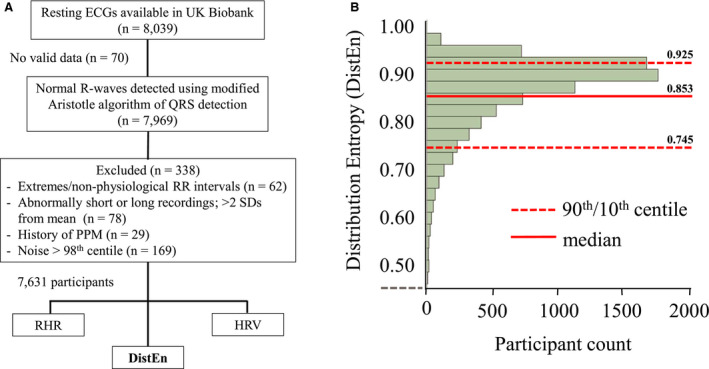
Flowchart of analytical approach and distribution of entropy (DistEn) distribution. **A**, Analytic approach for R‐wave selective and preprocessing of resting ECGs. **B**, DistEn with median (solid line) and 90th/10th percentiles (upper and lower dashed lines, respectively) by participant count. HRV indicates heart rate variability; PPM, permanent pacemaker; and RHR, resting heart rate.

Resting ECG data collection lasted 2 minutes with the patient in a seated position. Preprocessing steps are summarized in Figure 1A. Under resting conditions, 4 ECG electrodes were placed on the right and left antecubital fossa and wrist; a 3‐lead (leads I, II, and III) ECG recording (AM‐USB 6.5, Cardiosoft v6.51) at 500 Hz was taken during 2 minutes at rest and stored in xml format by Cardiosoft. Normal R waves were detected using the modified Aristotle algorithm of QRS detection.^22^, ^23^ RRIs >2s or <0.428s [RHR in beats per minute [bpm] <30 or >140] recordings (n=62) were excluded from analysis. The majority of records tightly coalesced around the mean duration of 117 seconds (SD=6 seconds). We excluded a small number of records (n=78) that were >2 SDs from the mean length as they were found to have multiple gaps and/or excess noise. In keeping with prior studies using UKB ECG records,^24^, ^25^ we further removed recordings with artifacts. Noise level was determined by calculating the overall SD of a moving SD with a window length of 3 beats; an overall SD close to zero indicated minimal to no noise, whereas the 98th percentile of SDs was considered excessive and excluded (n=169). We excluded an additional 29 patients who were known to have had a pacemaker insertion procedure before ECG assessment. In total, we inspected 1 in 20 raw ECG tracings or RRI profiles at random to ensure quality control and appropriate RRI detection. Data from 7631 participants were entered for analysis (Figure 1A).

### RHR and Time‐Domain HRV Analysis

RHR in bpm was calculated as the inverse of the mean RRI in milliseconds multiplied by the number of milliseconds per minute (60×1000) for each recording. Using established criteria, the root mean square of successive differences (RMSSDs), SD of NN intervals, and percentage >5, 10, and 20 milliseconds for differences between adjacent RRIs were calculated as measures of time‐domain HRV.^26^, ^27^

### DistEn for Heartbeat Complexity Analysis

A host of entropy measures for assessing physiological complexity have been established in the past decade; conceptually, they all share the same theoretical basis—the system is more complex (or unpredictable) if new information in its output (usually a time series) occurs at a higher rate. To assess such evolution of information in a time series, data are first reconstructed to a higher dimensional state space that is believed to best characterize the dynamic changes in the temporal structure of the fluctuations. A state, defined by a vector in the state space, renders new information if it appears far enough in distance from other states that already appear within the time series. With a threshold parameter for distance, approximate entropy and sample entropy essentially measure the log conditional probability that such a new state emerges.^28^

For traditional entropy measures, data length of 10 minutes to hours have been used.^9^, ^12^, ^29^, ^30^ If time series were short (eg, 2 minutes in this study), not even a single state would meet this stringent threshold criterion, resulting in a zero probability, and therefore an invalid result. This is a well‐known limitation of parameter dependence where the number of states that survive the threshold criterion can vary widely depending on the threshold parameter chosen. DistEn was developed specifically to handle short time series.^13^, ^15^ It takes full advantage of between‐states distances by measuring the distribution pattern instead of hard‐coding them as within or beyond the threshold parameter. This strategy appears to be more nuanced because the distribution of the distance (but not the mean/median distance) clearly show different patterns among synthetic time series with different dynamical regimes.^13^ With its established statistical properties, and given the short length of these UKB resting ECGs, we decided to mainly utilize DistEn.

### Algorithm for DistEn

For an RRI time series *x*=*x*(*i*), 1≤*i*≤*N*, its *m*‐dimensional state space reconstruction can be obtained by:

(1) $$u_{m}\left(i\right)=\left\{x(i),x(i+\tau ),\ldots ,x(i+(m‐1)\tau )\right\},$$

where 1≤*i*≤*N*−m*τ*; *τ* and *m* represent the time delay parameter and the dimension parameter, respectively. The Chebyshev distance between a pair of states *u_m_*(*i*), *u_m_*(*j*) can be calculated by:

(2) $$d\left[u_{m}\left(i\right),u_{m}\left(j\right)\right]=\underset{0\leq k\leq m‐1}{\text{max}}\left(|x(i+k\tau )‐x(j+k\tau )|,\;1\leq i,\;j\leq N‐m\tau \right).$$

Next, a histogram approach with a fixed bin number *B* is used to estimate the empirical probability density function of the distance matrix $d[u_{m}\left(i\right),u_{m}\left(j\right)],1\leq i,j\leq N‐m\tau$ except the main diagonal (i.e., *i*≠*j*). Using {*p_t_*, *t*=1,2,…,*B*} to denote the probability of each bin, DistEn is defined by the following formula:

(3) $$\text{DistEn}\left(m,\tau ,B\right)=‐\frac{1}{\log_{2}\left(B\right)}\sum_{t=1}^{B}p_{t}\log_{2}\left(p_{t}\right)$$

Further graphical representation of these methods are shown in Figure S1. The parameters used were as follows: *m*=3, *τ*=1, and *B*=256 based on our prior studies using similar data.^13^, ^14^, ^15^ We tested DistEn using a variety of parameters within recommendations and did not see meaningful effects on our results. Figure S2 presents simulation results showing the stability and consistency of DistEn results across data length and parameter selection. DistEn ranges theoretically between 0 and 1, with larger values indicating higher complexity. Figure S2A and S2B show ECG strips over 15 seconds for 2 participants with low and high DistEn but similar heart rate and mean RRI.

### Mortality Outcomes

Our primary outcome was all‐cause mortality obtained from death certificates within the UK National Health Service (NHS) Information Center and the NHS Central Register for Scotland. All death certificate details were provided directly to the UKB. Primary cause of death was classified using *International Classification of Diseases, Tenth Revision* (*ICD‐10*), codes by trained personnel. Secondary outcomes of cause‐specific mortality were grouped into cardiovascular‐ (I00‐I99), respiratory‐ (J00‐J99), and cancer‐ (C00‐D48) related primary causes of death.

### Assessment of Covariates

Demographics included age, sex, ethnicity, and education. Age at baseline assessment was calculated in years based on their dates of birth. Sex (men/women) and ethnicity were self‐reported. Since the majority of participants self‐identified as British or “white” European descent (94%), we included ethnicity as European and non‐European. Education was college‐level (yes/no). For lifestyle factors, we included BMI (calculated as weight [kg] divided by height squared [m2]), alcohol use (<3 drinks per week, ≥3 drinks per week), and smoking status (never, former, or current), physical activity (summed metabolic equivalent minutes per week for all activities derived by Cassidy et al^31^). Socioeconomic status was assessed using the Townsend deprivation index (TDI), calculated immediately before the participant joined UKB based on the preceding national census output areas. Each participant was assigned a score corresponding to the output area in which their postcode was located.

Comorbidities relevant to mortality were based on self‐report and medications taken at baseline. These included cardiovascular diseases (CVDs), hypertension, high cholesterol, atrial fibrillation (AF)/arrhythmias, diabetes mellitus, angina, myocardial infarction (MI), and peripheral vascular disease. CVD risk was split into low (no risks factors) and high (≥3 risk factors) for comparison purposes. Other comorbidities indicative of health status included cancer (yes/no, in response to "has a doctor ever told you that you have had cancer?"), respiratory diseases (chronic obstructive pulmonary disease, asthma, sleep apnea, or pulmonary fibrosis), psychiatric history (depression, anxiety, bipolar, schizophrenia, or suicidal intent), neurological disorders (dementia, Parkinson disease, ischemic/hemorrhagic stroke, epilepsy, or multiple sclerosis head/spinal injuries), musculoskeletal disorders (osteoarthritis, rheumatoid arthritis, or other inflammatory arthropathies), gastrointestinal disorders (ulcers, liver disease, inflammatory bowel disease), renal disorders (kidney failure, dialysis, nephropathies, or pyelonephritis), endocrine disorders (thyroid, parathyroid, hypothalamic/pituitary, or adrenal disorders), hematological disorders (anemia, thrombocytopenia, hemophilia, sickle cell, or thalassemia), and number of medications taken.

### Statistical Analysis

The characteristics of patients who died versus those who survived during follow‐up were assessed using *t* tests for continuous variables and chi‐square tests for categorical variables. We determined the relationship between DistEn and dichotomized baseline variables (age >65 years versus ≤65 years), sex (men versus women), ethnicity (European versus non‐European), college attendance (yes versus no), BMI (>35 versus ≤35), TDI (fourth versus first quartiles), physical activity level (fourth versus first quartiles), CVD risk (high versus low), β‐blocker usage, AF/arrhythmias, and pacemaker presence using multivariable regression. Cox proportional hazards models were used to assess the predictive value of DistEn for all‐cause and cause‐specific mortality: (model A) a core model controlled for demographics; (model B) TDI and lifestyle factors; (model C) cardiovascular risks/diseases; (model D) other comorbidities; and (model E) final adjustment was made for autonomic measures (heart rate, MAP, and RMSSD). We also tested for interactions between DistEn and age, sex, and CVD risk factors. Hazards ratios (HRs) and corresponding 95% CIs represent a 1‐SD decrease in DistEn. The proportional hazards assumption was assessed using the global chi*‐*square test in R package cox.zph (survival) incorporating methods described by Grambsch and Therneau.^32^. Efron method was used to handle ties. All other statistical analyses were performed using JMP Pro (version 14, SAS Institute).

## Results

### Participant Characteristics

Baseline characteristics of 7631 participants (mean age, 59.5 years [SD, 7.6 years]; 4610 [60.4%] women) are summarized in Table 1 based on survival or death from any cause. During a median follow‐up of 7.8 years, the 451 participants (5.9%) who died were 3.3 years older at baseline (62.6 versus 59.3 years), more likely men (55% versus 39%), a current smoker (19.7% versus 11.9%), a former smoker (54.7% versus 47.7%), socioeconomically deprived (higher TDI, 0.03 versus −0.44), had lower college attendance (17.4% versus 25.5%), and had more comorbidities and medication usage (5.8 versus 3.8).

**Table 1:** Demographics, Lifestyle, and Clinical Comorbidities at Baseline

	Alive	Died	*P* Value
	(n=7180)	(n=451)	
	Mean (SD) or n (%)	Mean (SD) or n (%)	
Demographics			
Age at baseline, y	59.3 (7.7)	62.6 (5.9)	<0.0001
Men, %	38.6	55.0	<0.0001
College attendance, %	25.5	17.3	0.0001
Townsend deprivation index	−0.44 (3.2)	0.03 (3.3)	0.003
Ethnic background (European), %	86.7	92.3	0.0006
Lifestyle			
BMI, kg/m^2^	29.4 (5.9)	29.9 (6.3)	0.16
Smoking (current), %	12.1	20.1	<0.0001
Smoking (former), %	47.7	54.7	<0.0001
Physical activity (MET‐min)*	40.3 (0.3)	36.2 (1.0)	<0.0001
Alcohol (≥3 drinks per wk), %	32.4	32.2	0.90
Cardiovascular disease, %			
Hypertension	47.7	60.5	<0.0001
High cholesterol	18.8	23.1	0.02
Angina	8.9	15.5	<0.0001
Myocardial infarction	4.9	10.9	<0.0001
Diabetes mellitus	9.9	23.5	<0.0001
AF/arrythmias	2.5	3.8	0.09
Clinical comorbidities			
Medications taken	3.8 (3.4)	5.8 (4.2)	<0.0001
Cancer, %	9.7	26.4	<0.0001
Respiratory, %	30.2	38.8	0.0002
Gastrointestinal, %	9.7	9.4	0.93
Renal, %	1.6	0.4	0.05
Endocrine, %	6.4	4.7	0.14
Musculoskeletal, %	7.6	13.2	0.09
Psychiatric	17.1	18.9	0.34
Neurological, %	7.5	15.7	<0.0001
Hematological, %	1.6	3.1	0.011
Derived ECG metrics			
DistEn	0.856 (0.08)	0.819 (0.11)	<0.0001
RRI, ms	865 (148)	834 (170)	<0.0001
RHR, bpm	71.2 (12.4)	73.7 (15.0)	<0.0001
RMSSD**	2.59 (0.65)	2.36 (0.79)	<0.0001
MAP, mm Hg	105.6 (15.3)	104.5 (15.5)	0.17

UK Biobank participant characteristics at baseline expressed as mean (SD) for continuous variables or percentage for presence of categorical variables. Participants were compared based on survival status (alive/died). Categorical data are presented as percentage of participants present. *P* values from 1‐way ANOVA tests for continuous measures and Pearson chi‐square tests for categorical data. Physical activity: summed metabolic equivalent (MET) minutes per week for all activities. AF indicates atrial fibrillation; BMI, body mass index; bpm, beats per minute; DistEn, distribution entropy; MAP, mean arterial pressure; RHR, resting heart rate; RMSSD, root mean square of successive differences between normal heartbeats; and RRI, RR interval.

* Square root transformation.

** Log‐transformed.

### DistEn, Baseline Comorbidities, and Cardiovascular Risk

The median value for DistEn was 0.853 (0.877 [SD, 0.078]; range, 0.301–0.975) (Figure 1B). Differences in values for DistEn by patient characteristics and cardiovascular risk, adjusted for age, sex, ethnicity, and education, where appropriate, are summarized in Table 2. DistEn was moderately lower (ie, lower complexity) in those aged >65 years (effect size −0.16 SD, *P*<0.0001), men (−0.12 SD, *P*<0.0001), non‐Europeans (−0.18 SD, *P*<0.0001), low physical activity (lowest quartile; −0.20 SD, *P*<0.0001), and high socioeconomic deprivation (TDI) (highest quartile; 0.25 SD, *P*<0.0001). The difference in DistEn was larger in patients with BMI >35 (−0.41 SD, *P*<0.0001) and those with the highest cardiovascular risk (≥3 risk factors; −0.46 SD, *P*<0.0001). DistEn was marginally lower in those taking β‐blockers (0.07 SD, *P*=0.046), but was not different in those with previously reported AF/arrythmias (*P*=0.68).

**Table 2:** DistEn Differences by Patient Characteristics

	Present	Absent	Difference (SD)	*P* Value
	Mean (SD)	Mean (SD)		
Age >65 y	0.840 (0.002)	0.854 (0.001)	0.014 (−0.16)	<0.0001
Men	0.843 (0.002)	0.852 (0.002)	0.009 (−0.12)	<0.0001
European ancestry*	0.855 (0.001)	0.840 (0.003)	0.015 (+0.18)	<0.0001
College attendance	0.849 (0.002)	0.846 (0.001)	0.003 (+0.04)	0.10
BMI >35 kg/m^2^	0.819 (0.003)	0.852 (0.001)	0.033 (−0.41)	<0.0001
High deprivation**	0.861 (0.002)	0.841 (0.002)	0.020 (−0.25)	<0.0001
Low physical activity***	0.840 (0.002)	0.856 (0.002)	0.015 (−0.20)	<0.0001
High CVD risk****	0.822 (0.003)	0.859 (0.002)	0.037 (−0.46)	<0.0001
β‐Blocker usage	0.843 (0.003)	0.849 (0.001)	0.006 (−0.07)	0.046
AF/arrhythmias	0.850 (0.006)	0.848 (0.001)	0.002 (+0.03)	0.68
Pacemaker	0.852 (0.008)	0.848 (0.001)	0.004 (+0.05)	0.64

The difference in mean distribution entropy (DistEn) values and expressed in SDs by the presence/absence of covariates (adjusted for age, sex, ethnicity, and education from body mass index [BMI] onwards). AF indicates atrial fibrillation.

* European vs non‐European ancestry.

** Townsend deprivation index (TDI) fourth and first quartiles.

*** Metabolic equivalents–minutes first vs fourth quartile.

**** Cardiovascular disease (CVD) risk was determined as the sum of the binary covariates hypertension, cholesterol, diabetes mellitus, current smoker, and/or angina or myocardial infarction at baseline; if ≥3, CVD was considered high and if zero, CVD was considered low.

### Lower DistEn Independently Predicts Incident All‐Cause Mortality

DistEn was associated with all‐cause mortality using our core Cox proportional hazards model adjusted for demographics (age, sex, ethnicity, and college education) (Table 3; model A). Specifically, for each 1‐SD decrease in DistEn, the HR for mortality was 1.36 (95% CI, 1.26–1.46; *P*<0.0001], equivalent to the effect of being 5.5 years older at baseline. After adjusting for lifestyle factors (BMI, deprivation level [TDI], physical activity, alcohol, and smoking), the HR for mortality remained similar (Table 2; model B) (HR, 1.31; 95% CI, 1.21–1.41 [*P*<0.0001]). DistEn remained predictive of mortality after further adjustment for CVDs/risk factors (Table 2; model C) (HR, 1.27; 95% CI, 1.17–1.37 [*P*<0.0001]) and multiple comorbidities (Table 2; model D) (HR, 1.24; 95% CI, 1.14–1.34 [*P*<0.0001]).

**Table 3:** Distribution Entropy of Resting Heartbeats and Risk for All‐Cause Mortality and CVD‐, Respiratory Disease–, and Cancer‐Related Mortality

Model DistEn*	All‐Cause	CVD	Respiratory	Cancer
A (Core)	1.36 (1.26–1.46) <0.0001	1.56 (1.29–1.86) <0.0001	1.73 (1.41–2.11) <0.0001	1.24 (1.10–1.41) <0.0001
B (A+lifestyle)	1.31 (1.21–1.41) <0.0001	1.43 (1.17–1.74) 0.0004	1.80 (1.46–2.21) <0.0001	1.22 (1.07–1.39) 0.003
C (B+CVD)	1.27 (1.17–1.37) <0.0001	1.37 (1.12–1.68) 0.003	1.74 (1.41–2.15) <0.0001	1.19 (1.04–1.36) 0.01
D (C+comorbidities)	1.24 (1.14–1.34) <0.0001	1.35 (1.10–1.65) 0.004	1.70 (1.38–2.11) <0.0001	1.19 (1.03–1.36) 0.01
E (D+ANS measures)	1.22 (1.10–1.35) <0.0001	1.46 (1.11 −1.91) 0.007	1.75 (1.35–2.26) <0.0001	1.06 (1.13–1.26) 0.55

Cox proportional hazards models. Model A is our core model adjusting for demographics (age, sex, education, and ethnic background). Model B additionally includes lifestyle covariates—alcohol usage, smoking, body mass index, Townsend deprivation index, and summed active metabolic minutes per week. Model C builds on model B by including cardiovascular risks/disease—hypertension, cholesterol, peripheral vascular disease, diabetes mellitus, chronic heart failure, prior myocardial infarction, and arrhythmias. Model D builds on model C by including comorbidities—neurological disease, respiratory diseases, cancer, psychiatric disease, gastrointestinal/hepatic disease, musculoskeletal disorder, endocrine disorders, and hematological disease. Model E builds on model D by including autonomic nervous system (ANS) function indicators—resting heart rate, root mean square of successive differences between normal heartbeats, and mean arterial pressure. CVD indicates cardiovascular disease.

* Results for 1‐SD decrease in distribution entropy (DistEn) are presented as hazard ratio (95% CI) and *P* value.

### Relationship Between DistEn, Autonomic Variables, and Mortality

**Figure 2:**
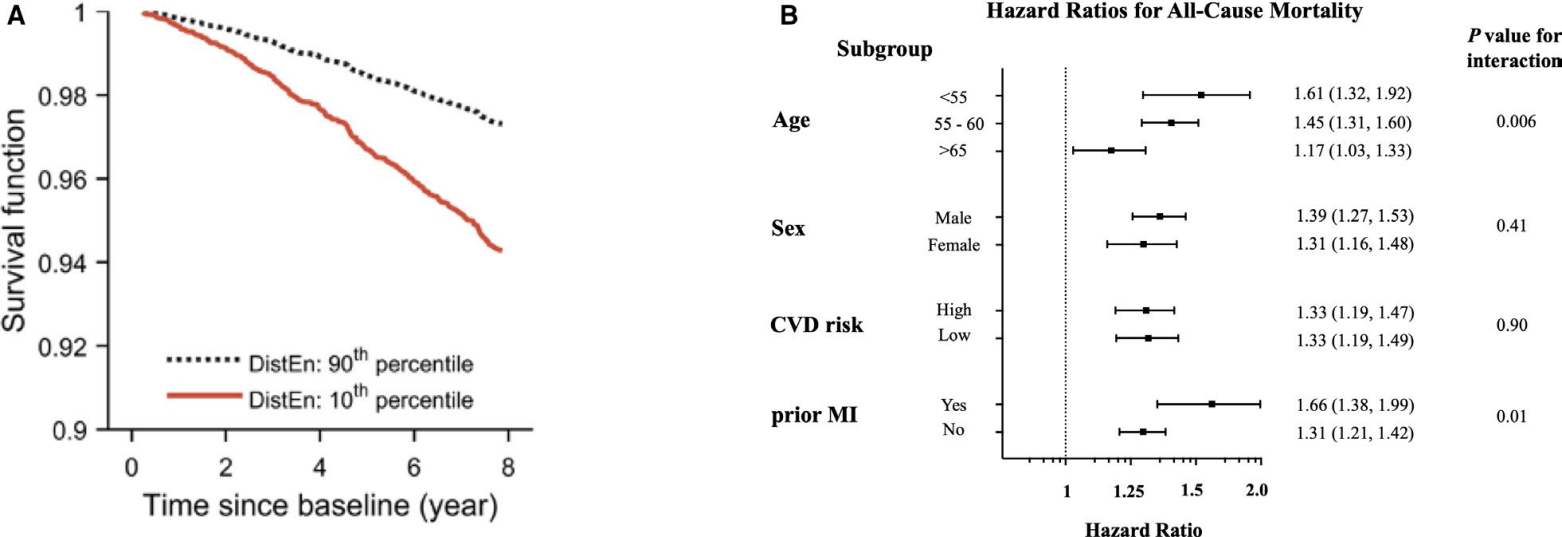
Survival function and subgroup analysis. **A**, Survival over time from Cox proportional hazards model for all‐cause mortality (model E: fully adjusted) based on those in the 90th (dotted line) and 10th (solid line) percentiles for distribution of entropy (DistEn) from 2 minutes of ECG at rest. **B**, Forrest plot of hazard ratios with 95% CIs for DistEn predicting all‐cause mortality based on subgroups of patients. Results presented on logarithmic scale. Cardiovascular disease (CVD) risk was determined high if there were ≥3 risk factors and low if there were no risk factors. Prior myocardial infarction (MI) as documented by date of *International Classification of Diseases, Tenth Revision (ICD)* diagnosis before UK Biobank assessment.

After controlling for autonomic‐related variables (RHR, RMSSD, and MAP), DistEn still remained predictive of mortality (Table 2; model E: HR, 1.22 [95% CI, 1.10–1.35], *P*<0.0001). Full model results are shown in Table S1. This was consistent regardless of which HRV variable was used. To put these results into context, we present fully adjusted model predictions for all‐cause mortality for participants with DistEn <0.745 (10th percentile) compared with DistEn >0.925 (90th percentile) in Figure 2A. The lowest decile had a 55% increased risk of all‐cause mortality compared with the highest decile; equivalent to being almost 9 years older at baseline, being a former smoker, or having diabetes mellitus at baseline (Table S1).

The mean RHR was 71.3 bpm (SD, 12.6) and significantly lower in those taking β‐blockers (58 versus 66 bpm, *P*<0.0001). We found DistEn to be negatively correlated with RHR (*r* ^2^=0.11, *P*<0.0001) and positively correlated with RMSSD (*r* ^2^=0.36, *P*<0.0001), but had no relationship with MAP (*P*>0.05). A 10‐bpm increase in RHR (HR, 1.14; 95% CI, 1.06–1.22 [*P*=0.0002]), and a 1‐SD decrease in RRI (HR, 1.20; 95% CI, 1.09–1.32 [*P*=0.0002]) or RMSSD (HR, 1.22; 95% CI, 1.09–1.35 [*P*=0.0003]), were separately predictive of mortality, but none of these associations remained after inclusion of DistEn (all *P*>0.05). Table S2 shows model results with all time‐domain HRV measures. In a direct comparison between DistEn and RRI survival models (adjusting for age, sex, and education), DistEn was superior across follow‐up years. Figure S3C presents concordance index (c‐statistic) differences.

### DistEn and Cause‐Specific Mortality

Of 451 deaths, the following primary causes were seen: 13.3% (60) cardiovascular, 10.0% (44) respiratory, and 42.8% (193) cancer. In Table 3, the increased risk from low DistEn was highest in model A where the primary cause of death was cardiovascular‐ (HR, 1.56; 95% CI, 1.30–1.87 [*P*<0.0001]) or respiratory (HR, 1.73; 95% CI, 1.41–2.11 [*P*<0.0001]) disease–related; and remained after adjustment for confounders (model B–E, Table 3). DistEn was weakly predictive for cancer‐related deaths in our penultimate model (model D: HR, 1.19; 95% CI, 1.03–1.36 [*P*=0.01]), but this was fully accounted for by inclusion of RHR and HRV measures (model E: HR, 1.06; 95% CI, 0.52–1.27 [*P*=0.55]).

### Subgroup Analysis and Interactions With DistEn

We did find a significant interaction effect between DistEn and age (*P*=0.006 for interaction) and prior MI (*P*=0.01 for interaction; Figure 2B). For every 1‐SD decrease in DistEn in those aged <55 years, there was a higher risk for death (HR, 1.61; 95% CI, 1.32–1.92 [*P*<0.0001]) than those >65 years (HR, 1.17, 95% CI, 1.03–1.33 [*P*=0.02]). Similarly, those with a prior MI (HR, 1.66; 95% CI, 1.38–1.99 [*P*<0.0001]) had a larger risk from every 1‐SD decrease in DistEn than those with none (HR, 1.31; 95% CI, 1.21–1.42 [*P*<0.0001]). Taken together, these interactions represented a 56% enhanced risk when comparing those <55 years with a history of MI, versus those >65 years without prior MI.

## Discussion

To the best of our knowledge, this is the first large‐scale implementation of heartbeat complexity in beat‐to‐beat recordings to explore its link to incident mortality, after almost a decade of follow‐up. The key novel finding from this current study is the ability to derive prognostic information related to mortality using only a 2‐minute resting ECG with DistEn, independently of clinically relevant risk factors and autonomic indices such as RHR, HRV, and MAP; this has traditionally not been feasible using other complexity measures.^13^, ^15^, ^29^ In a direct comparison between DistEn and RRI Cox models (adjusting for age, sex, and education), DistEn was superior across follow‐up years. Moreover, DistEn was significantly more predictive of death in younger patients and in those who had already experienced an MI at the time of recording. The observed risks for mortality are clinically meaningful given that these independent findings are readily obtained from resting ECGs, where the effect sizes are comparable to being up to a decade older, having been a former smoker, or having diabetes mellitus.

We should note that complexity is an umbrella term that lacks a clear scientific definition. A variety of algorithms have been established in the past decades, each of which is believed to capture some aspects of this dynamical property. Entropy measures have been widely used in assessing physiological complexity, partially because of their capability in handling limited length data.^33^ However, it is generally accepted that many established entropy measures, in fact, quantitate the degree of randomness of a temporal process, which is related to, but is distinct from, complexity. Neither an increase in the randomness nor an increase in regularity necessarily implies an increase in the complexity. In outputs of healthy physiological systems, complexity is characterized by temporal structures that represent a certain optimal balance between variability (randomness) and order (regularity). To demonstrate the conceptual differences between randomness and complexity, a multiscale entropy analysis has been proposed to quantify the profile of entropy as a function of time scale.^34^ Although the method successfully explained the seemingly controversial higher entropy values in some diseased states, its application to clinical recordings has been limited because the method also requires longer data for properly profiling the entropy results. Taking full advantage of the state‐space characteristic of a temporal process, DistEn can reconcile this complexity‐randomness paradox by unravelling the spatial structure of the state space, thus being different from previously established single‐scale entropy analyses.^13^

The physiological interpretation of DistEn in heartbeat fluctuations may be partially linked to the autonomic control on the circulatory system. Supporting this hypothesis, we find that DistEn is correlated with RHR and HRV that are known to be influenced by the autonomic nervous system. In particular, RMSSD mostly reflects respiration‐related beat‐to‐beat changes that are measures of parasympathetic involvement in circulatory control, also referred to as respiratory sinus arrhythmia.^35^ However, at the 2‐minute level, RHR is likely influenced by both sympathetic and parasympathetic inputs, and the nonrespiratory sinus arrhythmia aspect may also contribute to beat‐to‐beat HRV. Therefore, the relationship between RMSSD and aging/disease may be more nuanced than simply a decline in parasympathetic function.^36^ This may be relevant to our observation that DistEn was lowest in patients with BMI >35 and high CVD risk profile, where there has been a substantial body of evidence supporting the role of the sympathetic/parasympathetic nervous system balance in obesity,^37^, ^38^ hypertension,^39^, ^40^ diabetes mellitus,^41^, ^42^ or a combination in metabolic syndrome.^37^, ^38^ The sympathetic predominance seen in CVD is likely only partially reflected in higher RHR and lower HRV, whereas DistEn’s independent predictive value may be indicative of broader mechanisms. For example, DistEn may capture aspects beyond autonomic nervous system activity such as structural^8^, ^43^ and/or electrophysiological properties^13^, ^15^, ^44^ of the heart, metabolic,^45^ or endocrine/neurohormonal factors.^46^, ^47^

Another interpretation of our results is that complexity, as quantified by DistEn, may be a marker of frailty; it may reflect declines in physiological reserve across multiorgan systems, thus increasing vulnerability to stressors. DistEn applied to short screening ECGs may provide additional information regarding cardiac stress reactivity above that provided by traditional measures of cardiac autonomic function. For example, it has been shown that time domain HRV reflects respiratory sinus arrythmia, which is predominantly mediated by cardiac vagal outflow at rest.^35^ In heart failure, myocardial infarction, and stroke, respiratory sinus arrhythmia is usually either blunted or absent. This may partially explain our observation that DistEn is most predictive of cause‐specific cardiovascular‐ and respiratory‐related mortality (Table 3). Interestingly, in keeping with prior studies,^48^, ^49^ RHR and HRV were individually predictive of cancer‐related mortality, as was DistEn (Table S2). However, only RHR remained predictive when all 3 measures were included in the same model. Given their shared overlap in reflecting autonomic nervous system output, further studies are needed to understand the role of these measures in cancer‐related mortality.

In an effort to understand DistEn in patient subgroups, clinical factors of interest that may influence its value were examined and taken into account. Unsurprisingly, RHR was significantly lower in patients taking β‐blockers even after adjustment for demographics, yet there was only a weak effect of β‐blocker usage on DistEn (Table 2). History of AF/arrythmias did not have a significant effect on DistEn. Taken together, this suggests that DistEn is relatively robust to the effects of medications and/or conditions that may affect heartbeats. However, it is unlikely that patients were actively in AF/arrythmia during the recording period given their paroxysmal nature.^50^ Further work is needed to examine the effects of pacemaker devices and chronic AF on the predictive ability of DistEn for mortality.

Our observation that lower resting heartbeat complexity confers a greater risk of death in younger participants (<55 years), and those having already experienced an MI, is also particularly intriguing. We do note that a similar pattern was also observed for RHR^48^ and HRV during a 2‐minute ECG strip analysis.^45^ For example, Goldberger et al^6^ noted in their large, prospective cohort of 30,000 that mortality risk was more pronounced in patients 59 years (similar cutoff to ours) and even in patients <38 years (relative risks between 1.11 and 1.27). Even though complexity is known to decrease with age, it remains unclear whether DistEn observations attenuate with age because of changes in biology or simply because of smaller numbers of older people in our cohort. Complexity changes in heartbeat fluctuations with aging may be masked by increased underlying diseases and medications taken,^51^ which was not fully accounted for in this study as well as many other entropy studies. Nevertheless, these observations may be further evidence that hidden mechanisms related to poor health are better captured by low DistEn in younger patients. We postulate that cardiac reactivity to stress, which has an adverse effect on future cardiovascular risk,^52^, ^53^ is perhaps better reflected by DistEn in an age group where there are fewer comorbidities, extrinsic influences of medication, or biological interactions with disease associated with the aging process that attenuate the signal. Those with potential heart damage (eg, post‐MI) may exaggerate this response and is better reflected by heartbeat complexity.

Others have begun to explore heartbeat complexity as a novel approach to assessing cardiac stress reactivity,^54^ but more remains to be done to verify these observations and before clinical applications either for screening or risk stratification of patients. For example, validating the DistEn measure with echocardiographic evidence for post‐MI structural changes to the heart may shed mechanistic light on why there is such a strong link to mortality. Given the interaction results, one testable hypothesis is that cardiac rehabilitation post‐MI in middle age can be tracked using serial DistEn measures alongside multimodal trackers such as motor activity,^55^ with the goal to maintain cardiovascular well‐being and increase life expectancy.

### Limitations

Despite the strengths of this study, several limitations exist. The interpretation of these results from a clinically meaningful and statistically robust standpoint must be taken. With a large sample size, it can lead to tight CIs, so we have chosen not to make reference only to *P* values, which become less informative in these situations. The resting ECGs are readily obtained and effect sizes are significant, but whether DistEn can be influenced to make a clinical meaningful impact has yet to be tested. While all participants were conducting similar activities in answering questionnaires before their protocolized ECG recordings, we cannot control for the physical and mental state of the participant, in response to the multitude of mental tasks and recall requested of the participants by the UKB.^18^, ^20^ For example, respiratory patterns are known to heavily influence cardiac dynamics.^56^ Patients excluded were of similar age and demographics but they did have slightly more risk factors than those who were included per ECG criteria. This was, however, a relatively small portion (4.2% or 338/7969). While we cannot definitively exclude the possibility that other hidden differences existed in the patients excluded, we are reassured that, if anything, inclusion of participants with potentially slightly more risk factors would likely strengthen the signal from DistEn for mortality, given that DistEn was significantly different in groups separated by the presence/absence of risk factors related to mortality (Table 2). Using the primary cause of death is most definitive, but may miss other contributing diseases that lends caution when interpreting results of the cause‐specific models. Finally, the UKB is a single population of mostly Caucasian of European descent, limiting the generalizability of these results to certain parts of the world.

## Conclusions

Resting heartbeat complexity from short, resting ECGs was independently associated with mortality in middle‐ to older‐aged adults. These risks appear most pronounced in middle‐aged patients with prior MI, and may uniquely contribute to routine mortality risk screening. Our findings have the potential to be scaled‐up to remote monitoring in smartwatch applications of beat‐to‐beat heart activity and opens up a new avenue of research for resting heartbeat complexity as a vulnerability marker for stress reactivity.

## Sources of Funding

This work was supported by the National Institutes of Health (grant numbers T32GM007592 and R03AG067985 to L.G. and RF1AG059867, RF1AG064312, and R01AG048108 to K.H.), the Foundation of Anesthesia Education and Research (MRTG‐02‐15‐2020‐Gao to L.G.) and the BrightFocus Foundation (A2020886S to P.L.). UKB is generously supported by its founding funders the Wellcome Trust and UK Medical Research Council, as well as the Department of Health, Scottish Government; the Northwest Regional Development Agency; and the British Heart Foundation and Cancer Research UK.

## Disclosures

Scheer has received lecture fees from Bayer HealthCare, Sentara HealthCare, Philips, Vanda Pharmaceuticals, and Pfizer Pharmaceuticals. The remaining authors have no disclosures to report.

## Supporting information


**Tables S1–S2**
**Figures S1–S3**
