# Supplementary material for: Resting Heartbeat Complexity Predicts All‐Cause and Cardiorespiratory Mortality in Middle‐ to Older‐Aged Adults From the UK Biobank
Source: J Am Heart Assoc. 2021 Jan 19;10(3):e018483. doi: 10.1161/JAHA.120.018483 (PMC7955428; doi:10.1161/JAHA.120.018483)
Supplement: Supplementary file 1 — Tables S1–S2 Figures S1–S3 [file JAH3-10-e018483-s001.pdf]

SUPPLEMENTAL MATERIAL

Table S1. Full Cox proportional hazards models for DistEn and all-cause mortality.

	**Model 1<br>Core**	**Model 2<br>Lifestyle**	**Model 3<br>CVD**	**Model 4<br>Comorbidities**	**Model 5<br>Autonomic Function**
	HR (95% CI)<br>*p*-value	HR (95% CI)<br>*p*-value	HR (95% CI)<br>*p*-value	HR (95% CI)<br>*p*-value	HR (95% CI)<br>*p*-value
**DistEn [a]**	**1.36 (1.26 - 1.46)<br><0.0001**	**1.31 (1.21 – 1.41)<br><0.0001**	**1.27 (1.17 – 1.37)<br><0.0001**	**1.24 (1.14 – 1.34)<br><0.0001**	**1.22 (1.10 – 1.35)<br><0.0001**
Age [b]	1.06 (1.04 – 1.08)<br><0.0001	1.07 (1.05 – 1.08)<br><0.0001	1.06 (1.05 – 1.08)<br><0.0001	1.05 (1.03 – 1.07)<br><0.0001	1.05 (1.03 – 1.07)<br><0.0001
Male	1.81 (1.50 – 2.18)<br><0.0001	1.69 (1.39 – 2.06)<br><0.0001	1.53 (1.25 – 1.87)<br><0.0001	1.67 (1.36 – 2.04)<br><0.0001	1.69 (1.39 – 2.07)<br><0.0001
European	1.48 (1.04 – 2.12)<br>0.023	1.51 (1.03 – 2.21)<br>0.034	1.60 (1.09 – 2.34)<br>0.008	0.79 (0.61 – 1.01)<br>0.06	0.78 (0.60 – 1.00)<br>0.05
Education (College)	0.69 (0.54 – 0.89)<br>0.002	0.73 (0.57 – 0.94)<br>0.016	0.76 (0.59- 0.98)<br>0.033	1.46 (1.00 – 2.14)<br>0.04	1.47 (1.02 – 2.17)<br>0.04
BMI (kg/m^2)		0.99 (0.98 – 1.01)<br>0.497	0.99 (0.97 – 1.00)<br>0.075	0.99 (1.00 – 1.01)<br>0.12	0.98 (0.97 – 1.00)<br>0.09
Townsend Deprivation Index		1.04 (1.01 – 1.07)<br>0.016	1.04 (1.00 – 1.07)<br>0.024	1.03 (1.00 – 1.06)<br>0.06	1.04 (1.01 – 1.07)<br>0.02
Physical Activity		0.99 (0.99 – 1.00)<br>0.0004	0.99 (0.99 - 1.00)<br>0.001	0.99 (0.99 – 1.00)<br>0.02	0.93 (0.91 – 0.97)<br>0.02
Smoking (Current)		2.13 (1.59 – 2.86)<br><0.0001	2.11 (1.58 – 2.84)<br><0.0001	2.07 (1.54 – 2.78)<br><0.0001	2.00 (1.51 – 2.63)<br>0.0001
Smoking (Former)		1.49 (1.19 – 1.88)<br>0.0006	1.43 (1.14 – 1.80)<br>0.002	1.42 (1.13 – 1.79)<br>0.003	1.43 (1.13 – 1.80)<br>0.003
Alcohol (>3 Drinks per Week)		0.89 (0.68 – 1.16)<br>0.337	0.94 (0.72 – 1.23)<br>0.668	1.07 (0.81 – 1.40)<br>0.89	1.02 (0.82 – 1.25)<br>0.89
Hypertension			1.04 (0.84 – 1.29)<br>0.726	0.89 (0.71 – 1.12)<br>0.32	0.89 (0.71 – 1.12)<br>0.34
Cholesterol			0.86 (0.69 – 1.09)<br>0.207	0.78 (0.62 – 0.98)<br>0.13	0.78 (0.62 – 0.98)<br>0.03

	Model 1<br>Core	Model 2<br>Lifestyle	Model 3<br>CVD	Model 4<br>Comorbidities	Model 5<br>Autonomic Function
	HR (95% CI)<br>*p*-value	HR (95% CI)<br>*p*-value	HR (95% CI)<br>*p*-value	HR (95% CI)<br>*p*-value	HR (95% CI)<br>*p*-value
Peripheral Vascular Disease			1.40 (0.52 – 3.80)<br>0.506	1.52 (0.56 – 4.11)<br>0.44	1.58 (0.58 – 4.27)<br>0.37
Diabetes Mellitus			1.86 (1.46 – 2.39)<br><0.0001	1.59 (1.23 – 2.06)<br><0.0005	1.54 (1.19 – 1.99)<br>0.0009
Chronic Heart Failure			2.97 (0.94 – 9.37)<br>0.064	3.01 (0.94 – 9.67)<br>0.11	2.84 (0.88 – 9.17)<br>0.08
AF/Arrhythmia			1.34 (0.82 – 2.19)<br>0.240	1.19 (0.73 – 1.95)<br>0.49	1.11 (0.67 – 1.84)<br>0.69
Myocardial Infarction			1.38 (1.00 – 1.90)<br>0.046	1.13 (0.82 – 1.57)<br>0.46	1.19 (0.86 – 1.65)<br>0.28
Respiratory Diseases				1.17 (0.96– 1.43)<br>0.12	1.14 (0.93 – 1.39)<br>0.20
Neurological Diseases				1.65 (1.27 – 2.14)<br><0.0004	1.59 (1.23 – 2.07)<br>0.0005
Cancer				2.92 (2.35 – 3.62)<br><0.0001	2.86 (2.31 – 3.54)<br><0.0001
Psychiatric Disorders				0.75 (0.58 – 0.98)<br>0.030	0.76(0.59 – 0.99)<br>0.04
Gastrointestinal Diseases				0.99 (0.72 – 1.36)<br>0.962	0.99 (0.72 - 1.36)<br>0.95
Musculoskeletal Disorders				1.05 (0.79 - 1.39)<br>0.748	1.06 (0.80 – 1.40)<br>0.67
Endocrine Disorders				0.73 (0.47 - 1.15)<br>0.161	0.70 (0.44 – 1.10)<br>0.12
Hematological Disorders				1.90 (1.10 – 3.27)<br>0.034	1.95 (1.13 – 3.35)<br>0.02
Number of Medications Taken				1.08 (1.05 – 1.11)<br><0.0001	1.08 (1.05 – 1.11)<br><0.0001

	Model 1<br>Core	Model 2<br>Lifestyle	Model 3<br>CVD	Model 4<br>Comorbidities	Model 5<br>Autonomic Function
	HR (95% CI)<br>*p*-value	HR (95% CI)<br>*p*-value	HR (95% CI)<br>*p*-value	HR (95% CI)<br>*p*-value	HR (95% CI)<br>*p*-value
RHR (10 bpm increase)					1.10 (0.99 – 1.25)<br>0.08
RMSSD (1 SD decrease)					1.01 (0.90 – 1.15)<br>0.80
MAP (1 SD increase)					1.05 (0.95 – 1.16)<br>0.35

Full Cox proportional hazards models; results presented as hazard ratio (95% confidence interval), and p value. Model A is our core model adjusting for demographics (age, sex, education, and ethnic background). Model B additionally includes lifestyle covariates - alcohol usage, smoking, body mass index, Townsend deprivation index and summed active metabolic minutes per week. Model C builds on model B by including CV risks/disease - hypertension, cholesterol, peripheral vascular disease, diabetes mellitus, chronic heart failure, prior myocardial infarction and arrhythmias. Model D builds on C by including comorbidities - neurological disease, respiratory diseases, cancer, psychiatric disease, gastrointestinal/hepatic disease, musculoskeletal disorder, endocrine disorders, and hematological disease. Model E builds on D by including ANS function indicators – RHR, RMSSD and MAP. *DistEn* distribution entropy, *HR* hazard ratio, *SD* standard deviation, *CI* confidence intervals, *CVD* cardiovascular disease, *ANS* autonomic nervous system, *RHR* resting heart rate, *bpm* beats per minute, *RMSSD* root mean square of successive differences between normal heartbeats. [a] per 1-SD decrease, [b] per 1 year increase, [c] per 1unit increase, [d] per 1 MET-min/week increase, [e] per 1 medication increase, and [f] per 1-SD increase.

Table S2. **Associations of RHR and HRV variables with all-cause and cause-specific mortality.**

	All-Cause Mortality	Cardiovascular Mortality	Respiratory Mortality	Cancer Mortality
	HR (95% CI) *p* value	HR (95% CI) *p* value	HR (95% CI) *p* value	HR (95% CI) *p* value
RRI [a]	1.20 (1.09 – 1.32) 0.0002	0.95 (0.73 – 1.24) 0.80	1.25 (0.94 – 1.66) 0.11	1.38 (1.19 – 1.59) 0.0001
RRI [a] (+RMSSD)	1.14 (1.02 – 1.26) 0.02	0.85 (0.63 – 1.14) 0.28	1.16 (0.83 – 1.62) 0.38	1.33 (1.13 – 1.57) 0.0008
RRI [a] (+DistEn)	1.11 (0.98 – 1.20) 0.08	0.80 (0.60 – 1.06) 0.12	1.04 (0.77 – 1.42) 0.78	1.33 (1.13 – 1.56) 0.0005
RHR [b]	1.14 (1.06 – 1.22) 0.0002	0.97 (0.80 – 1.17) 0.80	1.21 (0.98 – 1.49) 0.08	1.26 (1.13 – 1.40) 0.0001
RHR [b] (+RMSSD)	1.10 (1.02 – 1.19) 0.02	0.89 (0.72 – 1.10) 0.28	1.12(0.87 – 1.42) 0.38	1.23 (1.09 – 1.39) 0.0008
RHR [b] (+DistEn)	1.10 (0.99 – 1.21) 0.08	0.85 (0.69 – 1.04) 0.12	1.03 (0.82– 1.29) 0.78	1.23 (1.10 – 1.39) 0.0005
RMSSD[†, a]	1.22 (1.09 – 1.35) 0.0003	1.41 (0.96 – 2.05) 0.08	1.39 (0.98 – 1.98) 0.06	1.25 (1.06 – 1.48) 0.009
RMSSD[†, a] (+RHR)	1.14 (1.02 – 1.28) 0.02	1.38 (1.00 – 1.90) 0.05	1.36 (0.92 – 2.01) 0.13	1.08 (0.90 – 1.30) 0.41
RMSSD[†, a] (+DistEn)	1.01 (0.90 – 1.15) 0.80	0.97 (0.70 – 1.35) 0.87	0.91 (0.64 – 1.29) 0.60	1.17 (0.94 – 1.44) 0.16
SDSD [†, a]	1.19 (1.08 – 1.33) 0.0009	1.28 (0.97 – 1.69) 0.08	1.47 (1.03 – 2.11) 0.04	1.25 (1.06 – 1.49) 0.009
SDNN [†, a]	1.26 (1.14 – 1.39) 0.0001	1.33 (1.03 – 1.74) 0.03	1.72 (1.23 – 2.40) 0.001	1.23 (1.05 – 1.45) 0.008
pNN5 [†, a]	1.23 (1.13 – 1.35) 0.0002	1.30 (1.03 – 1.65) 0.03	1.45 (1.08 – 1.94) 0.01	1.22 (1.06 – 1.40) 0.005
MAP [c]	1.05 (0.95 – 1.16) 0.35	1.02 (0.77 – 1.36) 0.88	0.74 (0.52 – 1.05) 0.10	1.05 (0.90 – 1.22) 0.57

Cox proportional hazards for associations between RHR, time-domain HRV variables, and MAP with all-cause mortality and cause-specific mortality (cardiovascular, respiratory and cancer related mortality) using our fully adjusted Model E. [a]for 1-SD decrease. [b]for 10bpm increase. [c]for 1-SD increase. *SD* standard deviation, *HR* hazard ratio, *CI* confidence interval, *RRI* RR interval, *RHR* resting heart rate, *HRV* heart rate variability, *RMSSD* root mean square of the successive differences between normal heartbeats, *SDSD* standard deviation of successive RRI, *SDNN* standard deviations of NN intervals, *pNN5* percentage of successive normal cardiac interbeat intervals greater than 5ms, *MAP* mean arterial pressure. [†] log transformed.

Figure S1. Demonstration and further description of the DistEn algorithm.

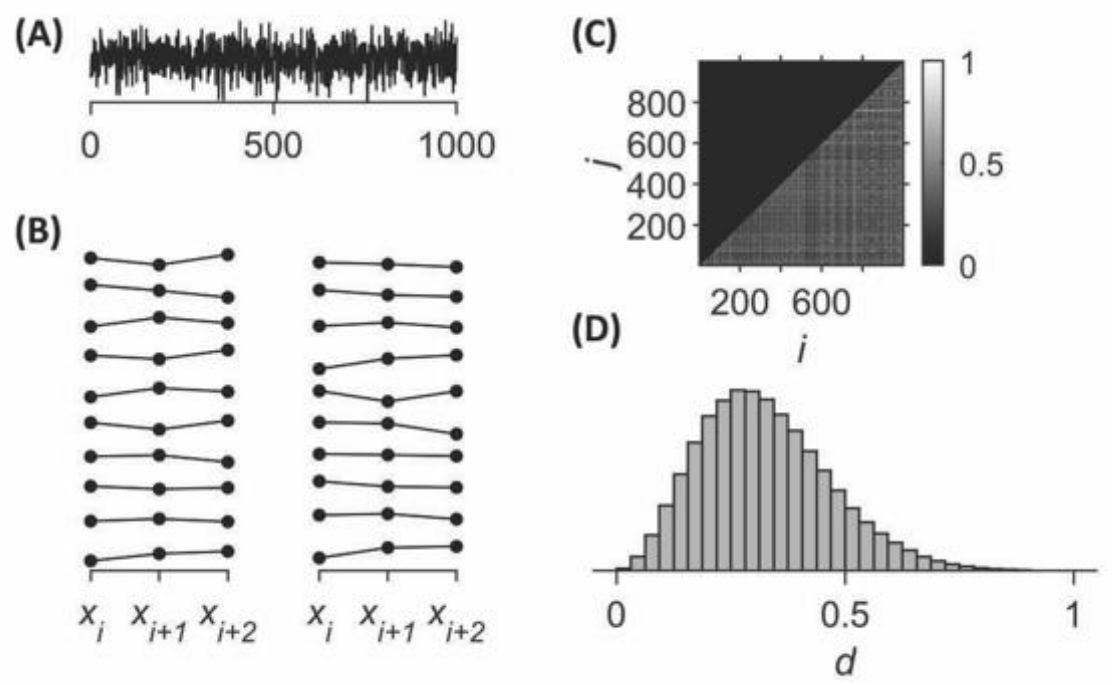

(A) An exemplary time series of 1000 points {x(i), 1 ≤ i ≤ 1000}. **(B)** The first 20 vectors **X**(i)={u(i),u(i+1),...,u(i+m-1)},1 ≤ i ≤ 20 in the state space after embedding state-space reconstruction. Here m indicates the embedding dimension which is set as 3. **(C)** The distance matrix **D**={$d_{i,j}$} with each element being the Chebychev distance among vectors **X**(i) and **X**(j) for all 1 ≤ i, j ≤ N − m, i.e., $d_{i,j}$ = max{|u(i + k) − u(j + k)|, 0 ≤ k ≤ m − 1}. **(D)** Probability density of $d_{i,j}$ estimated using histogram. If the histogram has B bins, we use p_t, t = 1, 2, ..., B to denote the probability (frequency) of each bin. To reduce bias, elements with i = j are excluded when estimating the ePDF. Then DistEn can be calculated by the Shannon entropy formula normalized by the theoretical maximum log(B).

Figure S2. DistEn simulation showing stability and consistency of results across data length and parameter selection.

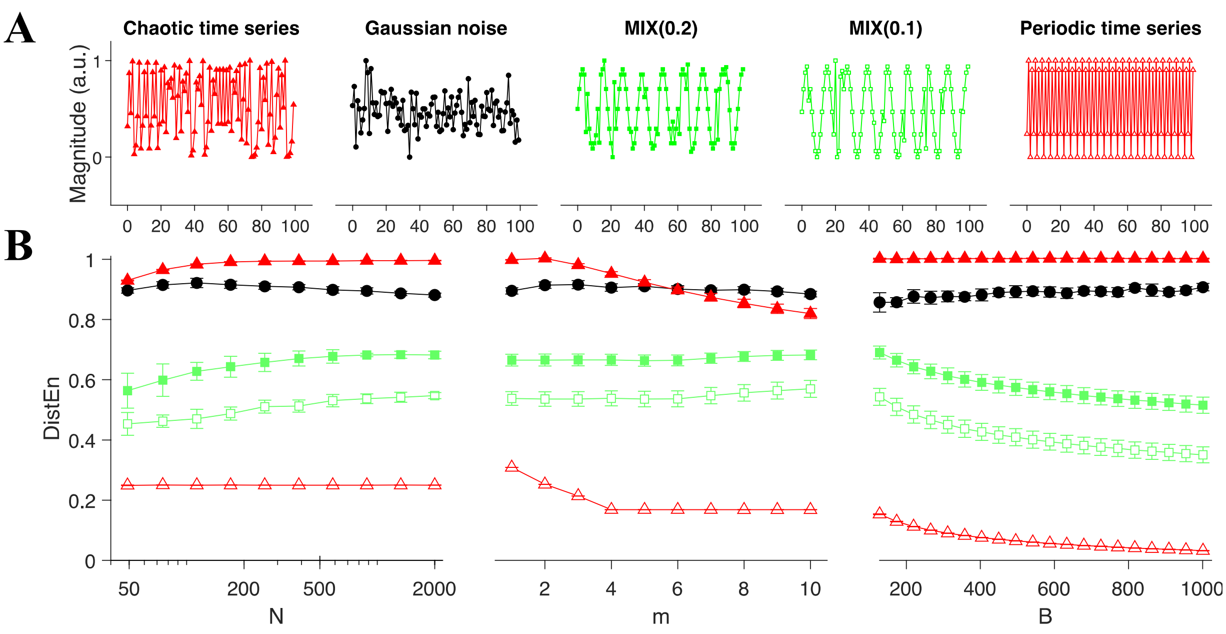

A: **Theoretical time series** with known complexity levels were generated to test the performance of DistEn. We simulated chaotic series, Gaussian noise, MIX(p) processes, and a periodic signal. Magnitude of signal in arbitrary units (a.u.) over time in seconds. The Logistic attractor $x(n + 1) = \omega \times x(n) \times (1 - x(n))$ was considered with $\omega = 4.0$ for chaotic series and $\omega = 3.5$ for periodic signals (period 4), respectively. The MIX(p) process is generated by a sinusoid signal of length N, with $N \times p$ randomly chosen points being replaced with independent identically distributed random noise. We applied p = 0.1 and 0.2, respectively, to generate two MIX(p) processes with different complexity levels. The Gaussian noise was generated by the random number function (randn) in MATLAB. **B**: **DistEn as a function of data length N (left panel)**, set at 10 values between 50 to 2,000, in the aforementioned five time series. *Error bars* indicates the standard deviation of 20 results. Note the relative stability i.e. narrow error bars of DistEn, and ability to separate all five signals across varying short signal lengths, *N*. **DistEn as a function of input parameter, *m* (middle panel)**: the dimension parameter (1 to 10). DistEn is consistent for MIX(p) signals and separate them well from other signals across the most commonly used 2, 3 or 4 values and beyond. **DistEn as a function of input parameter, *B* (right panel)**: the number of bins selected (128 to 1024) when *m* is held constant at 3. Again, note the relative consistency of results for DistEn, and ability to separate all five signals across varying number of bins selected, *B*.

Figure S3. ECG strips over 15s for two males with similar HR and mean RRI, and direct comparison of DistEn and RRI model performance

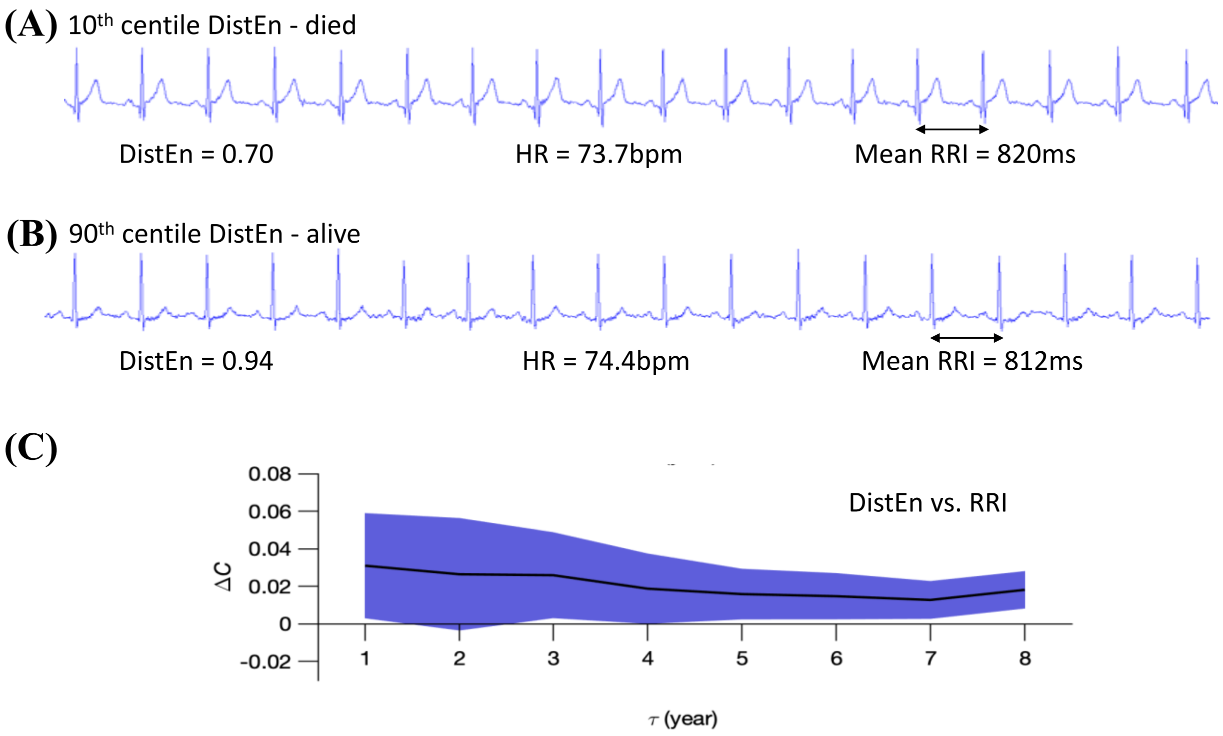

(A) Male subject within 10th centile DistEn who died during follow-up **(B)** Male subject within 90th centile DistEn who survived during follow-up. *DistEn* Distribution Entropy; *HR* heart rate; *RRI* RR-interval. Note the difference in the dynamical patterns is not visible to the naked eye, despite differences in nonlinear measures such as DistEn. **(C)** In a direct comparison between DistEn and RRI survival models (adjusting for age, sex and education), DistEn was superior across follow-up years. ΔC, difference in concordance index (c-stat), shaded region represents 95% confidence intervals after 100 iterations.
